# A Rare Case of Undifferentiated Rhabdoid Carcinoma of the Colon

**DOI:** 10.7759/cureus.31167

**Published:** 2022-11-06

**Authors:** Syed Alishan Nasir, Ronak Patel, Lalaine Ruiz, Michael Bush

**Affiliations:** 1 Internal Medicine, Norwalk Hospital, Norwalk, USA; 2 Pathology, Norwalk Hospital, Norwalk, USA

**Keywords:** rhabdoid phenotype, undifferentiated carcinoma, gastrointestinal perforation, colon cancer ck7, malignant rhabdoid tumor

## Abstract

Rhabdoid carcinoma of the colon is a rare type of malignancy that belongs to the family of malignant rhabdoid tumors (MRTs). This is infrequently encountered in clinical settings therefore data regarding treatment is lacking. Herein we discuss an elderly female patient who presented with severe abdominal pain in the setting of a gastrointestinal perforation. Imaging showed a large mass in the transverse colon, which was found to be an undifferentiated carcinoma of the colon with rhabdoid features. We highlight this case to discuss the clinical course of the patient, thereby adding to the limited literature that is available, as well as review the few studies that have, thus far, documented this rare presentation.

## Introduction

Undifferentiated carcinoma of the colon with rhabdoid features are tumors of the gastrointestinal (GI) tract infrequently reported in literature. These belong to the family of malignant rhabdoid tumors (MRTs) that are rare, associated with poor prognosis, and can occur in various organs including the GI tract, heart, nervous system, breast, and urinary tract [1,2]. Their histological hallmark consists of cells characterized by an eccentrically located and large nucleus, prominent nucleoli, and cytosolic aggregates of intermediate filaments [3]. On Immunohistochemistry, these tumors express both epithelial markers and mesenchymal markers. The first study that described this rare tumor pattern was published in 1978 by Beckwith and Palmer in which they reported an MRT in the kidney. It was therefore referred to as malignant rhabdoid tumor of the kidney (MRTK) [4]. This rhabdoid morphology has since been described to occur in epithelial tumors as well as within various mesenchymal and biphasic malignancies including synovial sarcomas [5]. Yang et al. in 1994 reported the first case of MRT of the colon [6]. Since then, a few reports have been published documenting this phenomenon, but rhabdoid carcinoma of the colon (RCC) remains a very rare occurrence with limited data on it.

## Case presentation

**Figure 1:**
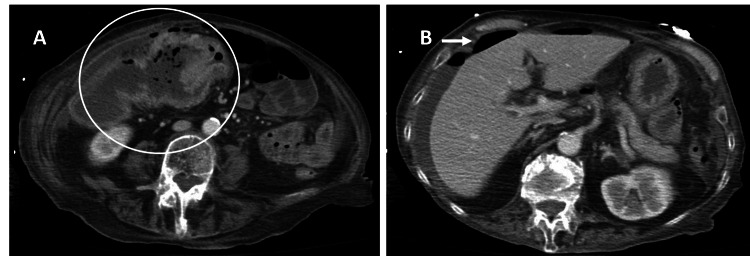
CT scan of the abdomen and pelvis showing (A) location of mass in the transverse colon (circle) and (B) the free air under diaphragm (arrow) concerning for perforation.

We present the case of an 81-year-old female with past medical history of hypertension, hyperlipidemia, and chronic obstructive pulmonary disease (COPD) who presented to the hospital with a two-day history of acute onset severe abdominal pain with radiation to the back. On initial evaluation, the patient was noted to be hypotensive with systolic blood pressures in the 80s mmHg, afebrile, and hypoxemic requiring 100% oxygen via a nonrebreather mask to maintain saturations in the 90s. She was noted to have a leukocytosis with a white blood cell count of 17×10^9^/L (normal range: <10×10^9^/L) and an elevated lactic acid level of 3.8 mmol/L (normal range: <2.2 mmol/L). Computed tomography scan of the abdomen and pelvis was obtained and showed a mass in the transverse colon with free air in the abdomen concerning for perforation (Figures 1A, 1B).

**Figure 2:**
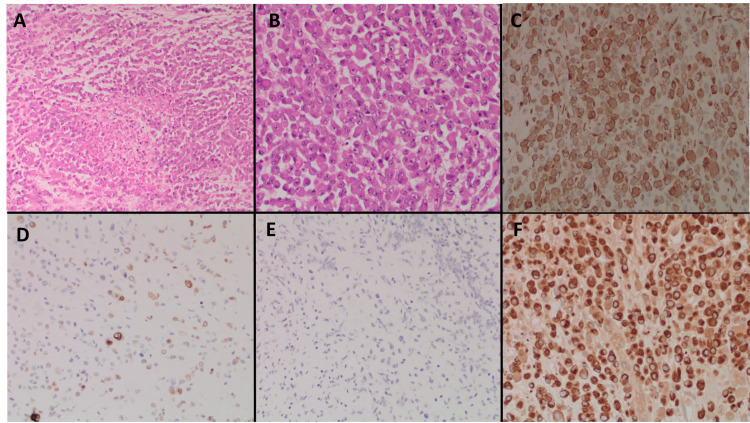
Pathology slides showing biopsy from the colon mass. The images show (A) 10x hematoxylin and eosin (H&E) stain showing rhabdoid features with central necrosis, (B) 20x H&E stain showing sheets and clusters of large epithelioid cells with vesicular nuclei, prominent nucleoli, multiple mitoses, and large paranuclear intracytoplasmic inclusions, (C) strongly positive vimentin stain, (D) cytokeratin (CK) 20 stain positivity, (E) CK7 stain negative, and (F) strongly positive CK AE1/3 stain.

Colorectal surgery was consulted, and the patient was taken to the operating room for exploratory laparotomy. The patient underwent extended right hemicolectomy with end ileostomy formation and an abdominal washout. Following surgery, the patient was admitted to the intensive care unit (ICU) as she required vasopressor support and was difficult to wean off the ventilator. Pathological evaluation of the patient's tumor demonstrated sheets of neoplastic discohesive cells with eosinophilic cytoplasmic inclusions (Figures 2A-2F). The nuclei were eccentrically located, variable in size, vesicular and were noted to have prominent nucleoli. No gland formation was seen and extensive tumor necrosis was present. Multiple mitoses were identified. Immunohistochemical stains showed that the tumor was strongly positive for cytokeratin (CK) AE1/AE3, CK CAM 5.2, and vimentin. Tumor cells were negative for smooth muscle myosin, desmin, CD138, C-kit, Melan A, and S100. Additional immunohistochemistry stains showed tumor cells positive for CK20 and negative for CK7 and CDX2. Sixteen lymph nodes were also evaluated which were negative for metastatic carcinoma. Immunohistochemistry was also performed for DNA mismatch repair proteins and showed loss of nuclear expression of MLH1 and PMS-2 but intact nuclear expression of MSH-2 and MSH-6. Genetic testing was performed and the patient was noted to have a BRAF1 mutation on exon 15 at codon 600. MLH1 methylation analysis demonstrated hypermethylation of the MLH1 promoter.

The rhabdoid morphology and staining pattern positive for epithelial markers (CK20, CKAE1/3) and mesenchymal markers (vimentin) supported the diagnosis of undifferentiated carcinoma with rhabdoid features. This was further confirmed by the presence of BRAF V600E mutation and hypermethylation of MLH1 promoter which confirmed this being sporadic cancer. Postoperatively, her course was complicated by atrial fibrillation with rapid ventricular response and superimposed aspiration pneumonia requiring multiple admissions to the ICU. Hematology/oncology was consulted and plan to start chemotherapy was deferred due to poor functional status. Patient and her family decided to opt for a comfort-based approach and no further treatment was administered.

## Discussion

RCC is an uncommon pathological entity that is almost always associated with an unfavorable prognosis, as are all MRTs. There has been some debate as to whether this is a phenotypical variant of MRT or an entity on its own. A literature review done by Moussaly and Atallah in 2015 studied all the cases of RCC documented since 1994. They found that RCC appeared to be a disease of the elderly with a mean age of 70 years at presentation and equal distribution between both sexes [7]. The most common complaint was abdominal symptoms such as abdominal pain, abdominal mass, and GI bleeding [7]. Out of the 17 cases studied by the authors, none were noted to have GI perforation which was a unique presentation in our patient. A second literature review was performed by Kojima et al. in 2021 where the authors reported a total of 28 cases from 1993 to 2019 [1]. Here, only four cases of RCC demonstrated no metastasis which was also consistent with our patient. Nonmalignant RCC therefore, appears to be more infrequent than malignant RCC. In the same study, the authors found that the median overall survival of patients with undifferentiated carcinoma with rhabdoid features was three months [1].

Malignant rhabdoid tumors (MRTs) are associated with carcinoma, sarcoma, and carcinosarcoma, depending on the tissue it develops in [1]. In colorectal adenocarcinomas, they are named as undifferentiated carcinomas with rhabdoid features or rhabdoid carcinoma of the colon. MRT cells have eosinophilic cytoplasm, oval or circular nuclei with prominent nucleoli, and vitreous inclusion bodies [1,3]. Tumor cells have low adhesion, high mitotic potential, and are prone to infiltrate other organs [1]. Although these tumors are morphologically sarcoma-like, immunohistochemical examination shows them to be negative for muscular markers and positive for epithelial (e.g., cytokeratin AE1/AE3) and mesenchymal cell markers (e.g., vimentin) [8]. MRTs of the colon are aggressive with high mortality, reported at 75% within the first six months following diagnosis [9]. The uncommon occurrence of malignant rhabdoid tumors (MRTs) has made it complicated to establish adequate survival-improving protocols [1]. Horazdovsky et al. concluded in their meta-analysis that surgery and actinomycin might improve survival in MRT [10].

MRTs are classified into two main categories termed “pure” or “composite”. The former is entirely composed of undifferentiated carcinoma cells whereas the latter consists of a combination of undifferentiated carcinoma cells and carcinoma cells with a high degree of differentiation [3,9]. In the setting of composite type of MRT, this is suggestive of rhabdoid features dedifferentiating from differentiated malignant cells [1,9]. In our patient, a pure type of RCC was seen as all cells appeared to be undifferentiated.

Genetic studies in our patient showed intact expression of MSH-2 and MSH-6, however, there was loss of expression of MLH1 and PMS-2. This is consistent with the study published by Remo et al. in which all neoplastic cells were observed to express human MSH-2 protein but were negative for human MLH1 [3]. We also found a BRAF V600E mutation and hypermethylation at the MLH1 promoter. Three separate investigators have searched for microsatellite instability, CpG island methylation, and KRAS & BRAF gene mutations in RCC. Remo et al. and Pancione et al. reported that their tumors harbored BRAF V600E mutations, high CpG island methylation, and wild-type KRAS phenotypes [3,11]. BRAF V600E mutation was also found in the case reported by Samalavicius et al. [5]. Another gene INI1 is a protein encoded by the tumor suppressor gene SMARCB1 and is expressed normally in the nucleus of cells, however, its expression is known to lack or be reduced in MRTs [1,12]. We did not investigate the expression of INI1 in this patient. 

## Conclusions

Undifferentiated carcinoma of the colon is infrequently encountered in clinical practice and data on its pathogenesis and treatment is limited. Our understanding mainly comes from isolated case reports and case studies due to which data surrounding it is lacking. It is a type of malignant rhabdoid tumor that is aggressive and associated with increased mortality. We present this case to highlight this rare finding and to add to the existing literature. We need further in-depth studies into this rare type of malignancy in order to identify better treatment options and improve mortality associated with it.
